# A high-generalizability machine learning framework for predicting the progression of Alzheimer’s disease using limited data

**DOI:** 10.1038/s41746-022-00577-x

**Published:** 2022-04-12

**Authors:** Caihua Wang, Yuanzhong Li, Yukihiro Tsuboshita, Takuya Sakurai, Tsubasa Goto, Hiroyuki Yamaguchi, Yuichi Yamashita, Atsushi Sekiguchi, Hisateru Tachimori, for the Alzheimer’s Disease Neuroimaging Initiative, Caihua Wang, Yuanzhong Li, Tsubasa Goto

**Affiliations:** 1 Imaging Technology Center, FUJIFILM Corporation, Kanagawa, Japan; 2 Department of Information Medicine, National Institute of Neuroscience, National Center of Neurology and Psychiatry, Tokyo, Japan; 3 Department of Psychiatry, Yokohama City University School of Medicine, Yokohama, Japan; 4 Department of Behavioral Medicine, National Institute of Mental Health, National Center of Neurology and Psychiatry, Tokyo, Japan; 5 Department of Clinical Epidemiology, Translational Medical Center, National Center of Neurology and Psychiatry, Tokyo, Japan; 6 Endowed Course for Health System Innovation, Keio University School of Medicine, Tokyo, Japan; 7 Imaging Technology Center, FUJIFILM Corporation, Kanagawa, Japan

**Keywords:** Risk factors, Alzheimer's disease

## Abstract

Alzheimer’s disease is a neurodegenerative disease that imposes a substantial financial burden on society. A number of machine learning studies have been conducted to predict the speed of its progression, which varies widely among different individuals, for recruiting fast progressors in future clinical trials. However, because the data in this field are very limited, two problems have yet to be solved: the first is that models built on limited data tend to induce overfitting and have low generalizability, and the second is that no cross-cohort evaluations have been done. Here, to suppress the overfitting caused by limited data, we propose a hybrid machine learning framework consisting of multiple convolutional neural networks that automatically extract image features from the point of view of brain segments, which are relevant to cognitive decline according to clinical findings, and a linear support vector classifier that uses extracted image features together with non-image information to make robust final predictions. The experimental results indicate that our model achieves superior performance (accuracy: 0.88, area under the curve [AUC]: 0.95) compared with other state-of-the-art methods. Moreover, our framework demonstrates high generalizability as a result of evaluations using a completely different cohort dataset (accuracy: 0.84, AUC: 0.91) collected from a different population than that used for training.

## Introduction

Alzheimer’s disease (AD) is a progressive disorder that causes brain cells to degenerate, and its symptoms, such as memory impairment, greatly impact the activities of daily living of affected patients. As of 2019, there were an estimated 5.8 million patients with AD, and this number is expected to grow to 13.8 million by 2050 in the USA alone^1^. On the other hand, AD has few available treatments, and there has been a high rate of failure in AD drug development programs^2^. New drug development remains challenging, and a large number of such drugs aim to slow the progress of AD at an early stage^3^. Participants with mild cognitive impairment (MCI) who are considered to be in the prodromal stage of AD are generally involved in clinical trials. For such trials, it is important to recruit participants with MCI who are likely to progress to AD, because only 20% of patients with MCI are subsequently diagnosed with AD within 1.5–2 years, while the other 80% remain unchanged or even revert to normal^4,5^. Therefore, an algorithm that can classify stable MCI (sMCI) and progressive MCI (pMCI) is needed to indicate that a patient with MCI will progress to AD within a certain period.

Various machine learning models for predicting the progress of AD have been proposed, and the most frequently utilized dataset, which includes brain images and cognitive test scores, is from the North American Alzheimer’s Disease Neuroimaging Initiative^6^ (NA-ADNI). Among these models, some^7–10^ use only brain images as input; the others^11–17^ use multimodal information including not only images but also cognitive scores. A summary of related works is provided in Supplementary Table 1, which shows that while some image-based methods have adopted an end-to-end approach, that is, using voxel-level images as inputs, almost all methods based on both image and clinical information use image features, such as hippocampus volumes, extracted by their own or existing open-source tools. Because most of these models are implemented using deep learning, which requires training of a considerable number of parameters, a large number of training samples is desirable to achieve good generalization performance^18^. Even though the classification accuracies of sMCI and pMCI have reached 75% to 86%, two problems have yet to be solved. The first problem is that the limited data in the NA-ADNI tend to induce overfitting. Compared with ChestX-ray^19^ and ImageNet^20^, which include samples from tens of thousands to millions of patients and objects, the NA-ADNI contains only about 1000 patients with MCI. The second problem is that, because of the participants’ biases, the actual ability of an artificial intelligence (AI) model trained and evaluated based on the NA-ADNI needs to be tested on a completely different dataset. One of the important biases is that the NA-ADNI participants are primarily enrolled in Caucasian populations^21^. Because differences in cortical structure have been reported between Caucasian and East Asian adults^22^, the performance of AI prediction models trained using the NA-ADNI should be examined using other cohort datasets. To the best of our knowledge, no such evaluations using cross-cohort datasets have been conducted.

The present study focuses on solving the above two problems based on information that can easily be obtained with relatively low invasiveness and cost, such as magnetic resonance imaging (MRI) images, which are used as brain imaging biomarkers; information such as positron emission tomography (PET) images is not adopted.

**Fig. 1:**
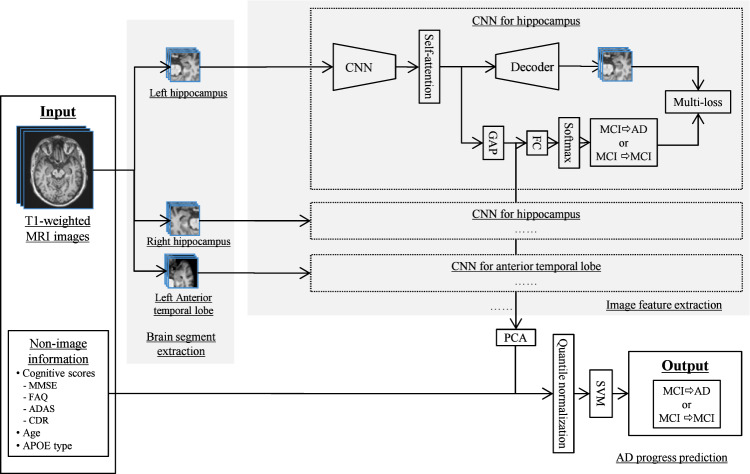
A machine learning framework for predicting the progression of Alzheimer’s disease. Multiple CNN-based image feature extractors with the same structure were trained on each brain segment, that is, the hippocampus and anterior temporal lobe in our study, to extract image features automatically. The CNN-based image feature extractor consists of a multi-layer convolutional neural network (CNN) block, a self-attention (SA) block, and an auto-encoder (AE) block. The feature vectors after the global average pooling (GAP) layer that encodes image features are passed to the PCA for dimensionality reduction. Finally, a linear SVM uses the low dimensional image features together with non-image information to make a final prediction.

Here, we propose a hybrid machine learning framework (Fig. 1) that consists of an image feature extraction part (IFEP) that automatically extracts image features from predefined brain sub-regions related to cognitive decline, and an AD progression prediction part (APPP), which predicts whether a patient with MCI will convert to AD using extracted image features combined with clinical information. In the IFEP, a convolutional neural network (CNN)^23^, which is a powerful tool used to extract features from images automatically^24^, was applied. In the APPP, a linear support vector machine (SVM) classifier was used to make the final predictions. In the IFEP, to suppress the overfitting of deep learning, we introduced prior brain imaging studies^25–27^ to use selected brain segments that have high correlations with cognitive decline to reduce the input image dimensions. To improve the performance and generalizability of our CNNs, mechanisms such as data augmentation^28^, dropout^18^, self-attention (SA)^29^, and auto-encoding (AE)^30,31^ were also used. A linear SVM tends to have higher generalizability compared with nonlinear classifiers^32^, and using it as a classifier for the image features extracted by CNNs has been shown to be effective for classification^33^. Therefore, in the APPP, we selected it for the final predictions using both the image features extracted in the IFEP and the non-image information, including cognitive scores, age, and genetic apolipoprotein E (APOE) types.

The contribution of this study is that we investigated a hybrid approach consisting of a deep CNN for automatic image feature extraction from cognitive decline-related segments and a linear SVM for final prediction using both automatically extracted image features and non-image information to tackle the problem of predicting MCI progress, which has traditionally been difficult in machine learning because of the lack of sufficient training data. Our study also showed that such a hybrid approach could achieve prediction accuracies of 88% (area under the curve [AUC]: 95%) for the NA-ADNI and 84% (AUC: 91%) for the J-ADNI, thereby outperforming previous works. The most important point of our study is that such a hybrid approach can be more practically applicable for predicting the progress of MCI because of its high generalizability for different cohorts.

## Results

### Training, validation, and test settings

**Fig. 2:**
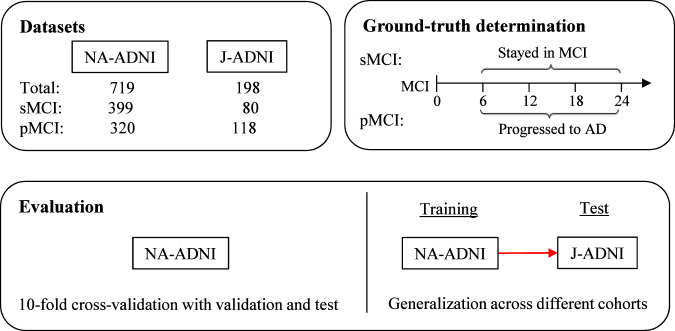
Training, validation, and test datasets. We extracted data for training, validation, and test from the longitudinal datasets ADNI1, ADNI-GO, and ADNI2. If a participant was diagnosed as MCI and both the MRI T1-weighted images and other non-image information used in our model were available, we selected the related data as a candidate for this study. We evaluated our model in the following two ways: first, as in previous studies, we tested the model trained based on the NA-ADNI dataset using repeated 10-fold cross-validation and test; in each iteration, 80% of the samples were used for training, 10% for validation, and 10% for test; second, to evaluate generalization across cohorts, we tested the model, which was trained based on the ADNI dataset using a totally “unknown” J-ADNI dataset.

The training, validation, and test datasets are shown in Fig. 2. Repeated 10-fold stratified cross-validation and test was adopted for evaluation of our model on the NA-ADNI dataset, and test was only carried out on the J-ADNI dataset that was completely unknown to the model. In the tenfold stratified cross-validation and test, the whole dataset was randomly split and stratified by class labels into 10 subsets. In each cross-validation and test, eight subsets were used for training; the remaining two were used for validation and test, respectively. The cross-validation and test were repeated 10 times by shifting the start subset of cross-validation and test setting to the next subset, so that each sample of all the datasets was used for test only once. The mean accuracy for all test subsets was calculated to evaluate our model in regard to the NA-ADNI dataset. For the evaluation of the J-ADNI dataset, the 10 models obtained in the above repeated 10-fold cross-validation and test were used, and the mean output probabilities of the models were used to classify each patient as sMCI or pMCI with a fixed threshold of 0.5.

### Comparison with previous studies

**Fig. 3:**
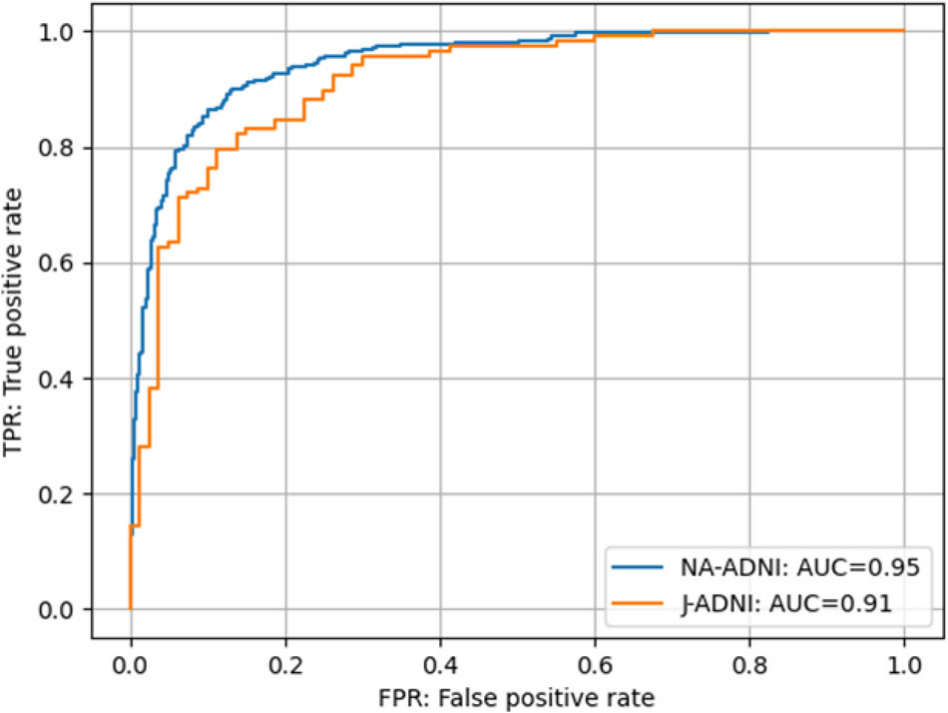
Cross-cohort evaluation results. ROC curves for both the NA-ADNI and J-ADNI are shown. The AUCs of the NA-ADNI and J-ADNI are 0.95 and 0.91, respectively.

A comparison of our results with those from previous studies is shown in Table 1. Because the same NA-ADNI dataset was used, the prediction performance was basically comparable, despite differences such as the follow-up periods used to define sMCI and pMCI, input information, and evaluation methods (validation, k-fold cross-validation, and k-fold cross-validation and test). For the case of using only brain images as input, our model (**M2** in Supplementary Table 2), based on only T1-weighted MRI images (accuracy: 78%, AUC: 85%, sensitivity: 78%, specificity: 78%), performed better than a previous deep neural network (DNN)^8^ (accuracy: 75%, AUC: not available [NA], sensitivity: 75%, specificity: 75%), equivalent to a DNN^9^ that used a much easier task definition (accuracy: 79%, AUC: NA, sensitivity: 75%, specificity: 82%), but was inferior to a DNN^7^ using MRI and PET images (accuracy: 83%, AUC: NA, sensitivity: 80%, specificity: 84%) and a DNN^10^ using mixed groups of cognitively normal (CN) + sMCI and pSMI+AD groups for training (accuracy: 83%, AUC: 88%, sensitivity: 76%, specificity: 87%). For the case of using not only images, but also non-image information, the performance of our model (**M5** in Supplementary Table 2) (accuracy: 88%, AUC: 95%, sensitivity: 88%, specificity: 88%) was better than that of state-of-the-art models using SVM^13^, DNN^16^, and random forest^17^ (accuracy: 85%–87%, AUC: 87%–90%). Considering that the state-of-the-art methods were evaluated only by validation datasets (not test datasets), the potential superiority of our model should be greater (see Supplementary Table 6). The experimental results indicated that our framework outperformed previous models and worked well on cross-cohort datasets, as shown in Table 1 and Fig. 3.

**Table 1:** Comparison with previous studies.

Method	Dataset	Input	Period	Accuracy	AUC	Sensitivity	Specificity
DNN [7]	NA-ADNI	MRI, FDG-PET	3 years	0.83	–	0.80	0.84
DNN [8]	NA-ADNI^a^	MRI	3 years	0.75	–	0.75	0.75
DNN [9]	NA-ADNI	MRI	1.5 years^b^	0.79	–	0.75	0.82
DNN [10]	NA-ADNI	MRI	3 years	0.83^c^	0.88^c^	0.76^c^	0.87^c^
SVM [11]	NA-ADNI	MRI, Cognitive scores	3 years	0.85	–	0.47	0.97
Random forest[12]	NA-ADNI	MRI, gender, age	1 year	0.79	–	0.82	0.74
SVM [13]	NA-ADNI	MRI, MMSE	3 years^b^	0.85^d^	0.90^d^	0.84^d^	0.88^d^
DNN [14]	NA-ADNI^a^	MRI, Cognitive scores, age	Series	–	0.76	–	–
DNN [15]	NA-ADNI	MRI, Cognitive scores, CSF, demographics	1 year	0.81	–	0.84	0.80
DNN [16]	NA-ADNI	MRI, Cognitive scores, APOE, gender, age	1 year	0.86^e^	–	0.82^e^	0.88^e^
Random forest[17]	NA-ADNI	MRI, PET, Cognitive scores, APOE	3 years	0.87 ^f^	0.87 ^f^	0.86 ^f^	–
Proposed (M2)	NA-ADNI	MRI	2 years	0.78	0.85	0.78	0.78
Proposed (M5)	NA-ADNI	MRI, Cognitive scores, APOE, age	2 years	0.88	0.95	0.88	0.88

^a^Relatively small datasets compared with the NA-ADNI were also used for both training and evaluation.

^b^Follow-up periods used for sMCI are longer than that of pMCI (5 years in DNN [9] and 4 years in SVM [13]).

^c^Trained with mixed groups of NC+sMCI and pSMI+AD.

^d^twofold validation accuracy.

^e^Tenfold cross-validation accuracy.

^f^Tenfold cross-validation accuracy, much higher accuracy of 91% on a small hold out test set.

### Cross-cohort evaluation results

Because only the NA-ADNI dataset obtained primarily using Caucasian populations was used for training our AI model, a non-Caucasian dataset was preferred for testing cross-cohort performance. The J-ADNI^34^ is a multicenter, longitudinal observational study in Japan using an almost identical protocol to the NA-ADNI. As a result, J-ADNI participants are East Asian. By using J-ADNI as the test dataset, we evaluated the cross-cohort performance of our AI model. The receiver operating characteristic (ROC) curve evaluated using the J-ADNI is shown in Fig. 3, and the ROC curve of the NA-ADNI is also shown as a reference. The **M5** model (Supplementary Table 2) was used in both cases. Although the AUC of the J-ADNI was 0.91 and 4% lower than the NA-ADNI, the performance (Supplementary Table 3) was still comparable to those in the previous studies shown in Table 1, where the NA-ADNI was used as both a training and test dataset.

### Visualization of our framework

**Fig. 4:**
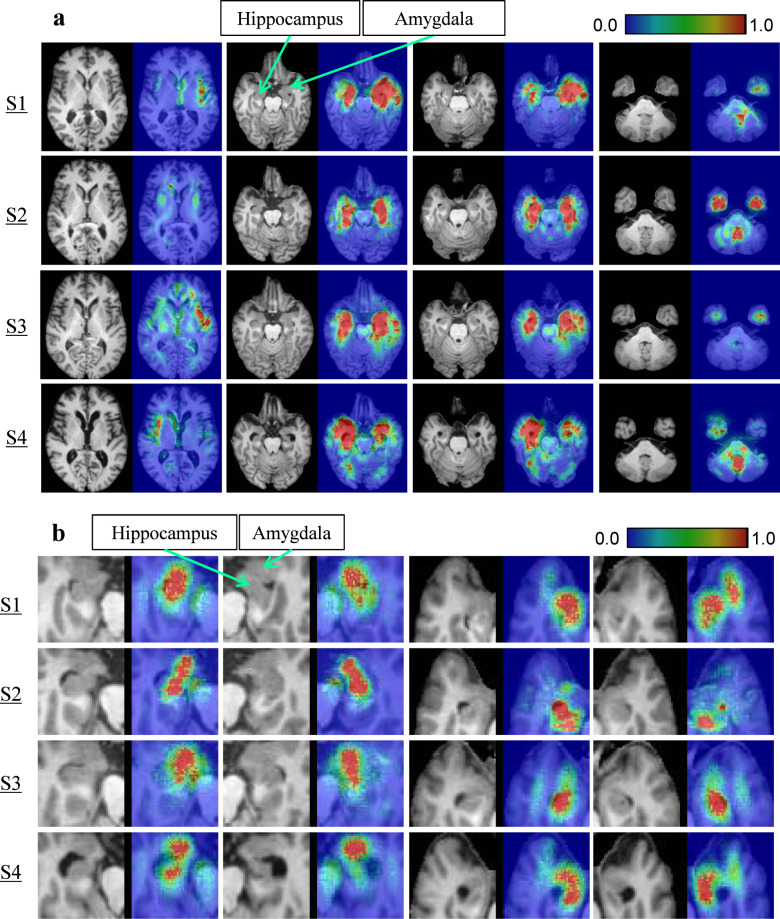
Gradient maps of CNNs. **a** Gradient maps of CNNs of the whole brain. CNN visualization results of M1 (Supplementary Table 2) based on the whole brain. Red represents a high contribution ratio, and blue represents a low contribution ratio. **b** Gradient maps of CNNs of brain segments. CNN visualization results of M2 (Supplementary Table 2) based on brain segments. Red represents a high contribution ratio, and blue represents a low contribution ratio. Compared with the whole brain, the CNNs based on brain segments consistently focused on the hippocampi and amygdalae.

**Fig. 5:**
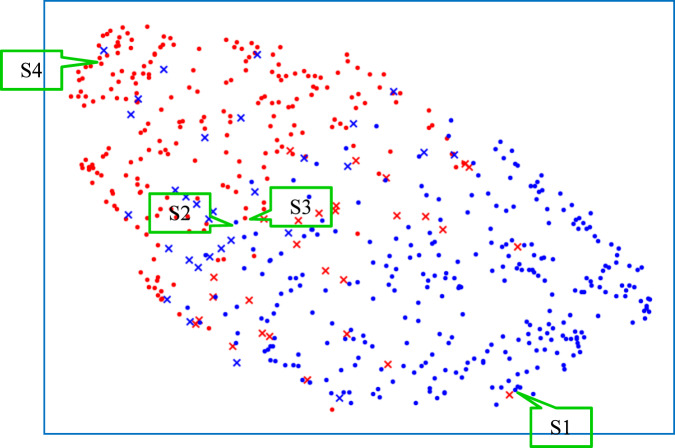
Visualization by UMAP (10 $\Rightarrow$ 2 dimensions). Training samples were plotted after the dimensions of their image and non-image features were reduced to 2 by using UMAP. Blue dots represent sMCIs that were correctly classified as sMCIs by our model, and blue “xs” represent sMCIs that were incorrectly classified as pMCIs. Red dots represent pMCIs that were correctly classified as pMCIs by our model, and red “xs” represent pMCIs that were classified incorrectly as sMCIs. Samples S1 to S4 were selected for CNN visualization in Fig. 4.

First, to investigate which region the CNNs focused on in the IFEP, SmoothGrad^35^ visualization was used. As shown in Supplementary Table 2, the performances using brain segments (**M2** and **M5**) were better than that using the whole brain (**M1 and M4**). The reason for this is shown in Fig. 4, where red represents a higher contribution ratio and blue represents a lower contribution ratio. The samples from S1 to S4 were selected from Fig. 5. Figure 4a shows that, even though **M1** and **M4** (Supplementary Table 2) focuses on the hippocampi and amygdalae, which play major roles in learning and memory, the variance of the “red” locations is quite large. On the other hand, as shown in Fig. 4b, **M2** and **M5** (Supplementary Table 1), which are based on brain segments, consistently focus on the hippocampi and amygdalae compared with the whole brain.

Second, to investigate the effectiveness of the features extracted by our model (**M5** in Supplementary Table 2), both the image and non-image features of the training samples were projected onto a two-dimensional (2D) space using UMAP^36^ (Fig. 5). Because we wanted to see the global structure in the features, the value of the parameter “n_neighbors” was set to 50, which is bigger than the default value of 15. The input for UMAP involves 10-dimensional features obtained just after the “Quantile normalization” shown in Fig. 1. The 10 dimensions consist of four from the hippocampi and anterior temporal lobes (left and right), four from different cognitive scores, one from age, and one from APOE type. A blue “•” represents sMCI, which was correctly classified as sMCI by our model, and a blue “x” represents sMCI, which was wrongly classified as pMCI. A red “•” represents pMCI, which was correctly classified as pMCI, and a red “x” represents pMCI, which was wrongly classified as sMCI. Despite a small number of misclassification errors, most training samples were appropriately distributed, which suggests that effective features for classifying sMCI and pMCI were extracted successfully, and that a linear SVM is appropriate for classification tasks.

## Discussion

One main challenge of this study is how to extract meaningful features from images effectively by using a CNN with a limited number of training samples. CNNs are considered a powerful method that can automatically extract more effective features from images than manually designed. To realize this, CNNs require a huge dataset for training a large number of parameters. However, in the present study, while the number of available samples was relatively small (in the hundreds), the dimension of input images for each sample is huge (millions of voxels). In this case, a CNN can easily find a local optimal solution for its parameters. As a result, trained CNN models with local optimal solutions tend to lack the generalization of unknown datasets.

To extract robust image features from limited data, we introduced prior knowledge of brain imaging studies that investigated the correlation between brain segments and the progression of AD. In the IFEP, instead of one CNN for the whole brain, multiple CNNs were trained for different brain segments cropped from the whole brain images. Hippocampi and anterior temporal lobes (left and right) were finally selected experimentally. By adopting the brain segments, the dimension of input images for each sample was reduced to less than one-tenth of the whole brain. Moreover, each CNN was forced to learn image features from only the regions that were highly correlated with the progression of AD. The gradient maps shown in Fig. 4 demonstrate that the image features extracted by the CNNs of the cropped segments focus on more meaningful regions, such as hippocampi and amygdalae, compared with the whole brain. In our experiment, as shown in Supplementary Table 2, adding image features extracted from the whole brain (**M4**) improved the accuracy of only using non-image information (**M0**) by 2%, and using image segments (**M5**) instead of the whole brain improved the accuracy further by 1%.

The other challenge is what kind of classifier should be used to make a final prediction based on not only the features extracted in the IFEP but also the non-image information, including cognitive scores, age, and genetic APOE type. As described previously, the visualized feature distribution of sMCI and pMCI shown in Fig. 5 suggested that a linear classifier was appropriate. In the APPP, to restrain the overfitting induced by the limited data, instead of an E2E deep neural network, a linear SVM^32^, which tends to have higher generalizability, was selected. The experimental results, in which the SVM model (**M5**, Supplementary Table 2) achieved 5% higher prediction accuracy than did the E2E model (**M3**, Supplementary Table 2), demonstrated the effectiveness of introducing the SVM.

In addition, our framework selected information that is relatively easy to be obtained as input. For example, even though PET imaging and cerebrospinal fluid (CSF) examinations are very useful for AD diagnosis, they were not selected because of their high cost and invasiveness. Furthermore, because the total processing time for predicting one case is about 30 seconds on a CPU-based PC, the hurdle to integrate our AI model into a practical clinical trial is low.

Successful and failure cases selected from the same UMAP visualization as that in Fig. 5 are shown in Supplementary Fig. 1. The “score” with a range from 0.0–1.0 is the output of the SVM; “0.0” and “1.0” represent the highest probability of sMCI and pMCI, respectively. The threshold for classification is 0.5. The samples S1–S6 shown in Supplementary Fig. 1a were selected from the cluster of sMCI samples, the boundary between the sMCI and pMCI clusters, and the cluster of pMCI samples.

S1 and S5 were selected from the cluster of sMCI samples. S1 is an sMCI case and was correctly classified as sMCI with a very low score of 0.15. S5 is a pMCI case and was classified wrongly as sMCI with a very low score of 0.09. Hippocampus atrophies of S5 are somewhat more progressive than S1, and cognitive scores are also slightly worse. However, because the score of S5 is lower than S1, our AI model shows that S5 has a lower probability to progress to AD. Note that the older population tends to have more progressive degradation of both hippocampus atrophies and cognitive scores caused by normal aging. The lower score of S5 might be affected by the fact that the age of S5 is 15 years older than that of S1.

S2 and S3 were selected from the boundary between the sMCI and pMCI clusters. S2 is an sMCI case and was classified correctly as sMCI with a low score of 0.30. S3 is a pMCI case and was classified correctly as pMCI with a score of 0.55, which is slightly higher than 0.5. Hippocampus atrophies of both S2 and S3 were unremarkable. On the other hand, the cognitive scores of S3 were slightly worse than those of S2. The difference in cognitive scores might have caused the different classification results.

S4 and S6 were selected from the cluster of pMCI samples. S4 is a pMCI case and was classified correctly as pMCI with an extremely high score of 0.95. S6 is an sMCI case and was classified wrongly as pMCI, also with a high score of 0.97. Regarding hippocampus atrophy, S4 is a little progressive and S6 is very progressive. Regarding cognitive scores, both are poor. Both the obvious hippocampus atrophy and the poor cognitive scores indicate the reason S6 was misclassified as pMCI.

The strength of deep learning models, but also one of their vulnerabilities, is the ability to discern patterns in training data that humans cannot^37^. Sometimes, the vulnerability is striking, especially where there are biases in the training dataset. For example, unintended effects caused by cohort (North America and East Asia) and gender biases have been reported in gender recognition systems^38^. As mentioned previously, because our AI model was trained based on the NA-ADNI dataset, which has a relatively small number of samples, mainly Caucasian, it is necessary to evaluate the trained model using a completely different dataset. In the present study, we evaluated its performance using the J-ADNI dataset, which consists of East Asian samples and was totally unknown to our AI model. Figure 3 shows that the performance of **M5** (Supplementary Table 2) using the J-ADNI. Furthermore, as shown in Table 1 and Supplementary Table 3, the prediction accuracy using the J-ADNI was 84% and comparable to previous studies that used the NA-ADNI as both the training and test datasets. The high performance shows the potential of our AI model to be incorporated in a practical clinical trial. A previous SVM study^13^ (Table 1) also used the J-ADNI dataset for evaluation of their model trained on an NA-ADNI dataset, but the evaluation was only carried out for CN and pMCI subjects (accuracy: 91%, AUC: 97%), because their definition of sMCI required a follow-up period of over 4 years, but the longest follow-up period in the J-ADNI is 3 years.

Supplementary Table 4 shows the results of an ablation study of neural network architecture (that is, DenseNet with and without SA and AE). AE contributed the most in the NA-ADNI dataset, whereas SA contributed the most in the J-ADNI dataset. The final DenseNet model with both SA and AE showed stable performance for both the NA-ADNI and J-ADNI datasets.

Supplementary Table 5 shows the performance of an alternative model in which SVM linear kernel was replaced by radial basis function kernel. Compared with Supplementary Table 4, the two models were nearly equivalent, except that the AUCs of the linear model were slightly better, implying that the linear SVM is preferable.

Generally, an end-to-end deep learning classification mode often suffers from overfitting when the dataset is small, and replacing classification layers in the end-to-end model with a linear SVM can improve its robustness against overfitting. We investigated the difference in accuracies between the validation dataset, which was used to select the best parameters for a model, and the test dataset, to which the selected model was applied. The lower difference of the two accuracies suggests higher model generalizability. Supplementary Table 6 shows that the accuracy difference between the validation and test of hybrid model (**M5**) was much smaller than that of the end-to-end model (**M3**). A similar tendency was also seen in the models using images only (**M2** and **M6**). Supplementary Table 6 also shows that the accuracy varied widely among the randomly split validation and test subsets because the dataset was relatively small. This strongly implies the existence of bias when a small, randomly-selected evaluation subset is used, and repeated 10-fold stratified cross-validation and test is desirable to evaluate a developed model objectively.

The first limitation of this study is that, even though the ADNI dataset, which is the largest worldwide regarding AD, was used in this study, the size was still relatively small. Therefore, we proposed the hybrid model to suppress overfitting.

The second limitation is that, because we aimed at contributing to clinical trials, only the baseline data were used as input. To extend its application to common clinical diagnoses, where longitudinal data are usually available, there is room for improvement by utilizing not only the baseline data, but also the whole longitudinal data.

The third limitation is that our hybrid model showed superiority to end-to-end models using DenseNet as a backbone when the available dataset was limited. However, end-to-end models, including those using other state-of-the-art architectures such as vision transformer^39^ as backbones, should be continuously investigated, because projects like the ADNI are ongoing and datasets will become larger.

In this paper, we proposed a hybrid machine learning framework to overcome the lack of training samples in the field of AD progression prediction. The experimental results showed that it worked well, even on different cohorts. Utilizing this framework to stratify patients with MCI who have higher risk of progression to AD might improve the success rate in future clinical trials of drugs for treating AD. To achieve this goal, the most important future tasks are to conduct retrospective evaluations based on previous trial data involving drug efficacy and to demonstrate the benefits of integrating AI quantitatively. In addition, as recent studies have shown that new biomarkers such as plasma phospho-tau are also effective for prediction^40^, it is necessary to consider adding these biomarkers to input in future models.

## Methods

### Datasets used in the study

The data used for training, cross-validation, and test in our study were obtained from the ADNI1, ADNI-GO, and ADNI2 datasets in the NA-ADNI database (http://adni.loni.usc.edu/ADNI). The NA-ADNI is a cohort study launched in 2003 as a public–private partnership, led by principal investigator Michael W. Weiner M.D., and carried out across 55 research centers in the USA and Canada. The clinical coordination center of NA-ADNI established a network of clinical sites and developed a plan for the recruitment and retention of subjects, and furthermore prepared the final clinical protocol and informed consent, which is distributed to the sites for local institutional review board (IRB) approval^6^, Subjects were willing and able to undergo test procedures, including neuroimaging and follow-up, and written informed consent was obtained from participants^17^. Over 2000 participants with normal cognition and patients with MCI or AD were recruited for the present study. The first cohort, referred to as ADNI-1, consists of 800 individuals: 200 CN individuals, 400 with late MCI, and 200 with mild dementia. ADNI-GO, the second cohort, included about 200 additional individuals with early MCI. In ADNI-2, more participants at different stages of AD were recruited to monitor the progression of AD. ADNI-3, which is presently enrolling additional CN individuals and patients with MCI and dementia, was not included in our study, because no diagnosis information is currently available.

Longitudinal data for each participant were examined, and the samples used in our study were selected as follows. From the longitudinal data, we first identified candidate baseline points at which the participant was diagnosed as having MCI and both T1-weighted MRI images and other non-image information used in our model were available. The non-image information includes cognitive test scores on the Mini-Mental State Examination (MMSE), Functional Activities Questionnaire (FAQ), Clinical Dementia Rating (CDR) Sum of Boxes score, and Alzheimer’s Disease Assessment Scale Cog-11 (ADAS), APOE type, and demographic information, such as gender and age. If the participant progressed to AD within 2 years from the baseline point, we labeled that participant, in association with the T1-weighted MRI images and non-image information at baseline, as a pMCI sample; otherwise, as an sMCI sample. There were 399 sMCI and 430 pMCI samples extracted for the training, validation, and test of our prediction model in this experiment.

The same procedure was applied to the J-ADNI dataset, which was collected at the 38 participating clinical sites using the same protocol as that for the ADNI, but from population of East Asian adults and in a shorter period (3 years). Approval for the J-ADNI study protocol was obtained from the local ethics committees or institutional review committees at the 38 participating clinical sites, including the principal investigator’s site (The University of Tokyo). Informed written consent was obtained from all the participants at each clinical site^21^. In total, 80 stable and 118 progressive MCI samples were extracted from the J-ADNI dataset for the evaluation of our trained model.

Detailed information of the NA-ADNI and J-ADNI datasets is shown in Supplementary Table 7.

### Image shape normalization

The 3D T1-weighted MRI images used in our study were first transformed into Montreal Neurological Institute (MNI)^41^ space by aligning T1-weighted MRI images to atlas images (standard images) created in MNI space. The template images of the MNI152 NLIN 2009a atlas^42^ were used. To align robustly and accurately T1-weighted MRI images, which were obtained under protocols used in NA-ADNI or J-ADNI studies, to the template images of the atlas, which were created in MNI space, a coarse-to-fine approach containing landmark-based and image registration-based alignment steps was developed.

In the landmark-based alignment step, six locations in the brain with distinct local anatomic structures were selected as landmarks to be used for robustly aligning T1-weighted MRI images to the atlas template images. Landmarks specified manually for the atlas template images are shown in Extended Fig. 2. These landmarks are the left and right eyes, body of fornix (front), midbrain (center), 4th ventricle (center), and corpus callosum (top). A region-based CNN (R-CNN)^43^ was trained to detect the same landmarks from the T1-weighted MRI images. We trained the R-CNN with 290 cases collected from multiple datasets, including the NA-ADNI dataset, OASIS Brains dataset^44^, and locally obtained data. Landmarks of training images were specified manually under the supervision of a radiological technologist.

When landmarks of T1-weighted MRI images were detected by the trained R-CNN, we set the center point of each landmark detected from each T1-weighted MRI image to correspond with that of the same landmark manually specified in the atlas template image. Using six pairs of corresponding central points of the landmarks in the T1-weighted MRI and atlas template images, we identified a linear transform, including 3D rotation, translation, and scaling, by minimizing the total error between the transformed central points of the landmarks detected from the T1-weighted MRI images and that of the atlas template images as follows:

1 $$\mathop {\sum }\limits_{i = 1}^6 \Vert Q_i - (SRP_i + T)\Vert\mathop { \to }\limits^{yields} min$$

where $P_i$ and $Q_i$ are the central points of the *i*th landmark in the atlas template and T1-weighted MRI images, respectively. ${{{\mathrm{S}}}}$, ${{{\mathrm{R}}}}$, and ${{{\mathrm{T}}}}$ denote 3D scaling, rotation, and translation, respectively. We used a linear transform with 9 degrees of freedom, called a similarity transform, instead of an affine transform because we only wanted to normalize the location, orientation, and size of the T1-weighted MRI images, not change the internal detail anatomic structures.

In the image registration-based alignment step, the initial similarity transform obtained from the landmark-based alignment was further refined by using an image registration technique that utilizes mutual information as an image similarity metric^45–47^. In this study, a brain mask of the atlas template images, which is available together with the atlas, was also used to ensure that the image registration was only performed on the brain region. The image registration technique used in this study was implemented based on the open-source Insight Tool Kit (ITK) image registration framework^48^.

### Image intensity normalization

As T1-weighted MRI images acquired in different sites with different equipment may have different biases in terms of intensity distribution, the normalization of image intensity is an important pre-procession for many image analysis tasks, and many methods have been proposed in the past^49–52^. In the present study, we adopted a similar example-based intensity normalization approach that uses patches in the T1-weighted MRI images and atlas template images, which contain patches of the acquired and tissue contrasts desired^50^, but implemented it in a simpler way for robustness and computational simplicity.

Supplementary Fig. 3 shows an outline of our image intensity normalization algorithm. Using the similarity transform obtained above, the T1-weighted MRI images were transformed to shape-normalized images to align to the atlas template images. Then, an augmented example-based image intensity normalization was applied to the shape-normalized images, the details of which are described below.

To ensure that the corresponding patches in the shape-normalized and atlas template images used for intensity normalization contained the same tissue, we performed nonlinear image registration between the shape-normalized and atlas template images. Nonlinear image registration was also implemented based on ITK^48^, and used mutual information as the image similarity metric and B-spline as the image transform^47^. Similar to what was done in the shape normalization, the brain mask of the atlas template image was also used to ensure that the nonlinear image registration was only performed on brain regions.

Using the B-spline transform obtained in the nonlinear registration, the shape-normalized images were transformed nonlinearly to the atlas template images, called the warped images, as follows:

2 $$I_w\left( X \right) = I_n(T_B\left( {X;\varphi } \right))$$

where $I_n$ and $I_w$ denote the shape-normalized and warped images, respectively, and $T_B\left( {X;\varphi } \right)$ is the B-spline transform between the shape-normalized and atlas template images with parameters of ${\upvarphi}$.

In the warped images $I_w$, tissues in the normalized image $I_n$ were nonlinearly deformed to align to those in the atlas template images. Using the brain mask of the atlas template images, we calculated the minimal box that contained the brain region, and divided the minimal boxes of both warped images $I_w$ and atlas template images $I_a$ into 8 × 8 × 8 blocks. For each pair of blocks in the warped and atlas template images at the same location, we calculated intensity histograms for both blocks of the pair and normalized the histogram range of the warped image block to that of the atlas template image block. Again, the brain mask of the atlas template image was used to exclude the voxels outside of the brain region based on the intensity histogram calculation. We normalized the histogram range instead of the histogram itself^52^ because the portion of tissues of different types may change substantially among subjects, and normalizing the histogram itself could lead to over-normalization. On the other hand, the histogram range, the lower and upper bounds of which stand for the intensities of CSF and white matter, respectively, is more stable.

For each block $B_k$, let $u_k^a$ and $u_k^w$ be the upper bounds and $l_k^a$ and $l_k^w$ be the lower bounds of the intensity histogram of the block in the atlas template images $I_a$ and the warped images $I_w$. We defined a linear intensity transform for block $B_k$ as follows:

3 $${\acute{I}}_k^w = \frac{{u_k^a - l_k^a}}{{u_k^w - l_k^w}}I_k^w - \frac{{u_k^a - l_k^a}}{{u_k^w - l_k^w}}l_k^w + l_k^a \equiv a_kI_k^w + b_k$$

where $I_k^W,k = 1, \cdots ,N$ denotes the intensity of voxels in block ${{{\mathrm{k}}}}$ of the warped images $I_w$, and ${\acute{I}}_k^w$ is its normalized intensity.

To obtain a continuous intensity transform over the whole images, we regarded Eq. (3) as the intensity transform at the gravity center of each block $B_k$, that is, the gravity center of brain mask of block $B_k$, and then used a kernel function to expand $a_k$ and $b_k$, two parameters that represent the intensity transform, to the whole image continuously. Using the gravity center $\bar X_k^w$ of block $B_k$ and its intensity transform parameters $a_k$ and $b_k$, we calculated two continuous parameter maps ${{{\boldsymbol{a}}}}_{{{\boldsymbol{w}}}}\left( {X^w} \right)$ and ${{{\boldsymbol{b}}}}_{{{\boldsymbol{w}}}}\left( {X^w} \right)$ of the intensity transform using a kernel function, defined as follows:

4 $${{{\boldsymbol{p}}}}_{{{\boldsymbol{w}}}}(X^w) = \mathop {\sum }\limits_{k = 1}^N p_kG\left( {X,\bar X_k^w} \right)/\mathop {\sum }\limits_{k = 1}^n G\left( {X,\bar X_k^w} \right)$$

where *p* stands for parameters $a$ or $b$ and $X^w$ stands for the 3D coordinate of each voxel in the warped image $I_w$. ${{{\mathrm{G}}}}({{{\mathrm{X}}}},\bar X)$ is defined as ${{{\mathrm{G}}}}\left( {{{{\mathrm{X}}}},\bar X} \right) = \exp \left( { - \frac{\Vert{X - \bar X}\Vert}{{2\left( {s_x^2 + s_y^2 + s_z^2} \right)}}} \right)$, where $s_x$, $s_y$, and $s_z$ are the block sizes. N is the number of blocks. Using the parameter map, we can normalize intensities in the warped image $I_w$ voxel-wise.

As Eq. (4) was defined on the warped images $I_w$, to obtain the intensity transform on the shape-normalized images $I_n$, we back-projected the parameter map obtained on the warped images $I_w$ to the shape-normalized images $I_n$ using the inverse of the B-spline transform obtained in the nonlinear image registration.

5 $${{{\boldsymbol{p}}}}_{{{\boldsymbol{n}}}}(X^n) = {{{\boldsymbol{p}}}}_{{{\boldsymbol{w}}}}(T_B^{ - 1}\left( {X^n,{\upvarphi}} \right))$$

where $X^n$ stands for the 3D coordinate of each voxel in the shape-normalized images $I_n$ and *p* stands for parameters $a$ or $b$. By applying the back-projected intensity transform parameters to the shape-normalized images $I_n$, we can obtain an intensity normalization.

### Brain segment extraction

After the intensity normalization was carried out, a skull-stripping process was performed on the shape-normalized images to extract the brain region. To achieve this, we trained a V-net^53^ with four layers. The V-net was trained using the same dataset as that used for the landmark detection. The ground truth of the brain regions of the training data was obtained by manual editing under the supervision of the radiological technologist. The segmentation results were generated automatically by an image registration method, which further included diffeomorphic image registration^54^ implemented in ITK.

From the normalized (in shape and intensity) and skull-tripped image, brain segments of hippocampi (left and right) and anterior temporal lobes (left and right) were extracted. The locations of these segments were identified in the atlas template image manually and their sizes were fixed to 64 × 64 × 64 voxels, which was large enough to contain each segment of interest with a necessary margin in the normalized image. For each sample, four brain segments, which contained hippocampi and anterior temporal lobes, both left and right, at the same locations specified in the atlas template images, were extracted from its normalized image, respectively.

### Image feature extraction

A densely connected convolutional network (DenseNet)^55^, which has confirmed superiority over other network architectures for the same task on a different dataset^56^, was adopted as the backbone of our architecture for image feature extraction. As shown in Supplementary Fig. 4, in our architecture, the DenseNet backbone was enhanced by adding an SA layer and an AE to improve its stability on limited training samples.

Supplementary Table 8 shows the detailed architecture of our DenseNet backbone, which is basically the same as DenseNet-121^55^, where the 2D convolution and pooling layers were modified to 3D ones. The size of the first convolution layer was also changed to a smaller one because of the smaller size of the brain segment images.

Between the last dense block and the classification layer of the original DenseNet-121, an SA layer was inserted. While DenseNet is a method used to extract local relationships from images hierarchically, the SA highlights important positions in a feature map and helps to extract a global relationship. In our model, by adding an SA layer just after the DenseNet backbone, we could extract features representing both the local and global relationships.

Beside the classification task, in which an inputted brain segment was classified to sMCI or pMCI classes, another task of the AE, which recovers the imputed brain segment image from the image features, was added to the DenseNet backbone. By introducing the AE task, which was considered more robust than classification with a small number of samples, the robustness of the entire architecture was expected to be improved. For the decoder, we employed a simple five-layer network consisting of de-convolution and up-sampling layers, the details of which are shown in Supplementary Table 9.

To optimize the multi-task architecture, a mixed loss function of the two tasks was defined as follows:

6 $${{{\mathrm{Loss}}}} = \left( {1 - {\upalpha}} \right)L_{class} + \alpha L_{AE}$$

where $L_{class}$ represents the classification error function for prediction defined by cross-entropy loss, $L_{AE}$ denotes the AE error function defined by smooth L1 loss, and *α* is a hyper-parameter that controls the effect of the AE, which is set to 0.8 empirically.

The two networks with the same architecture shown in Supplementary Fig. 4 were trained for the brain segments of hippocampi and the anterior temporal lobe independently. Because the left and right segments of brain are basically symmetrical, images of the left hippocampus were flipped horizontally and then used with images of the right hippocampus to train the hippocampus network; this process was also applied to the anterior temporal lobe segment. The output of the global average polling of the trained network was extracted as image features and used with other non-image features for final classification, as described below.

The intensity of all images for training and testing were normalized to [0, 1] by a fixed maximum intensity of 400 in advance. Because the number of samples was small, we also augmented the training images before they were inputted to the network. The following augmentations were carried out for each training image:

1. A similarity transform with random rotations ranged from −2.0 to 2.0 degrees, scaling factors from 0.95 to 1.05, and translation from −4.0 to 4.0 voxels, around or along the x, y, and z axes, respectively.
2. A linear gray scale transform with a random slope from 0.95 to 1.05 and a random shift from −0.05 to 0.05, where the gray value of the images was normalized to [0, 1].

To avoid the influence of the outside border generated by the random similarity transform augmentation, the augmented images were cropped into a 48 × 48 × 48 voxel size, and the cropped images were used as the input for our network.

### Linear SVM classification

Because the number of training samples is limited in the machine learning field of AD progression prediction, we adopted a linear SVM instead of E2E deep learning as the final predictor to improve generalizability. After a 128-dimensional image feature was extracted from each brain segment by CNNs, principal component analysis was used to reduce the dimension to 1 for each segment. We also attempted to reduce the dimension to 2 or 3 for each brain segment, but there were almost no changes in accuracy. Then, non-image information, including cognitive scores (MMSE, FAQ, CDR, and ADAS), age, and APOE type, was combined with the reduced image features of the four segments as the SVM input. Here, we transformed APOE type to a value representing AD risk based on the following rules:^57^ for ε2/ε3, value = 0.6; for ε3/ε3, value = 1.0; for ε2/ε4 and ε4/ε2, value = 3.2; and for ε4/ε4, value = 11.6. Because the distributions of these multimodal inputs differ greatly, quartile normalization^58^ is used to normalize the combined features before the SVM is applied. For validation and test, the same quartile normalization used for training was applied to the combined features.

### End-to-end (E2E) classification

We also implemented two E2E models using the same backbone architecture shown in Supplementary Fig. 4; one uses only images for prediction and the other uses both images and non-image information, as shown in Supplementary Fig. 5. For the model that uses both image and non-image information, bilinear fusion^16^ was used to combine the image features extracted from images and non-image features extracted from non-image information. The same AE as shown in Supplementary Fig. 4 was also adopted, but this was omitted because of space limitations. In the architecture, two image feature extraction modules were configured in parallel to extract image features from the images of the hippocampi and anterior temporal segments, and the extracted image features were concatenated together. For the model using both image and non-image information, the non-image features were first embedded and then extended to the same dimension of the image features. Bilinear fusion was then applied to the image features and extended non-image features to generate the multimodal features for classification.

In our experiments, although the E2E model achieved high validation accuracy, a relatively large decline in test accuracy (from 87% to 83%) was observed. On the other hand, only a slight decline in test accuracy was seen (from 89% to 88%) for our proposed architecture, which combined image and non-image features with a linear SVM, while it achieved the highest validation accuracy. This observation may imply that a simpler combination of multimodal features may be more stable than an E2E deep learning approach when the number of samples available is relatively small.

### Reporting summary

Further information on research design is available in the Nature Research Reporting Summary linked to this article.

## Supplementary information

Supplementary Meterials

Reporting summary

## Data Availability

The data used for model training, validation, and test are publicly available at the following URLs: 1 NA-ADNI dataset: http://adni.loni.usc.edu/. 2 J-ADNI dataset: https://humandbs.biosciencedbc.jp/en/hum0043-v1.

## Code availability

The DenseNet code, which was used as the backbone of our architecture, is available at https://github.com/liuzhuang13/DenseNet. The Faster R-CNN code used for brain landmark detection is available at https://github.com/rbgirshick/py-faster-rcnn. The Insight Toolkit: ITK, on which our image registration algorithm was implemented, is available at https://itk.org.
