# Supplementary material for: A high-generalizability machine learning framework for predicting the progression of Alzheimer’s disease using limited data
Source: NPJ Digit Med. 2022 Apr 12;5:43. doi: 10.1038/s41746-022-00577-x (PMC9005545; doi:10.1038/s41746-022-00577-x)
Supplement: Supplementary file 1 — Supplementary Meterials [file 41746_2022_577_MOESM1_ESM.pdf]

a

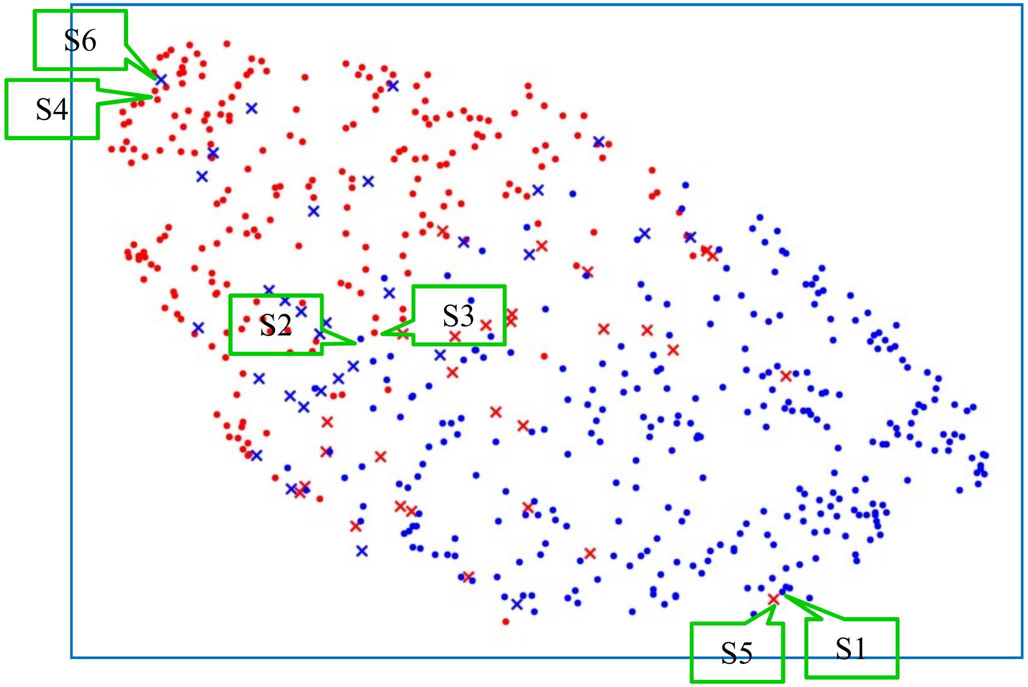

Supplementary Figure 1 Analysis of successful and failure cases selected from the UMAP plot. Supplementary Figure 1a: UMAP plot and selected samples. Training samples were plotted after the dimensions of their image and non-image features were reduced to 2 by using UMAP. Blue dots represent sMCIs that were correctly classified as sMCIs by our model, and blue “xs” represent sMCIs that were incorrectly classified as pMCIs. Red dots represent pMCIs that were correctly classified as pMCIs by our model, and red “xs” represent pMCIs that were incorrectly classified as sMCIs.

b

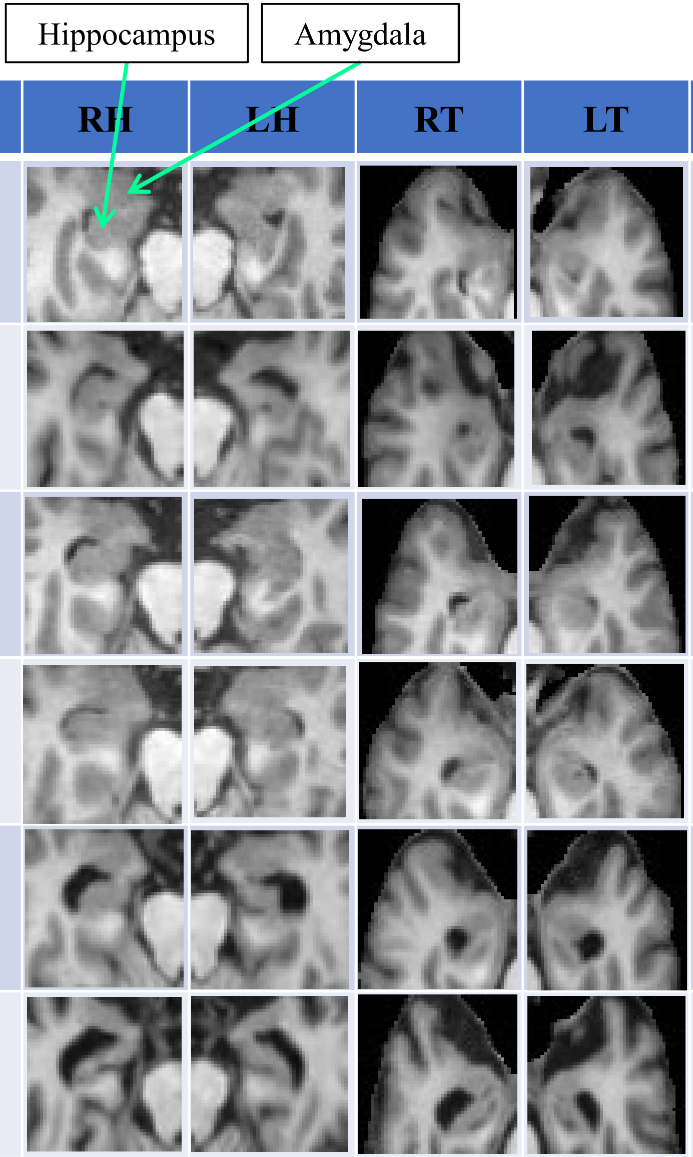

	Score	RH	LH	RT	LT	MMSE	FAQ	CDR	ADAS	Age	APoE
S1	**0.15**					**30**	**0**	**0.5**	**6**	**66**	**3×3**
S5	**0.09**					**26**	**0**	**0.5**	**9**	**81**	**3×3**
S2	**0.30**					**28**	**0**	**0.5**	**5**	**68**	**3×3**
S3	**0.55**					**29**	**0**	**1**	**7**	**68**	**2×3**
S4	**0.95**					**26**	**6**	**1**	**25**	**80**	**3×4**
S6	**0.97**					**26**	**13**	**2**	**15**	**80**	**3×3**

Supplementary Figure 1 Analysis of successful and failure case selected from the UMAP plot. Supplementary Figure 1b: Successful and failure cases of samples S1 to S6 selected for analysis from Supplementary Figure 1a. RH: right hippocampus; LH: left hippocampus; RT: right anterior temporal lobe; LT: left anterior temporal lobe.

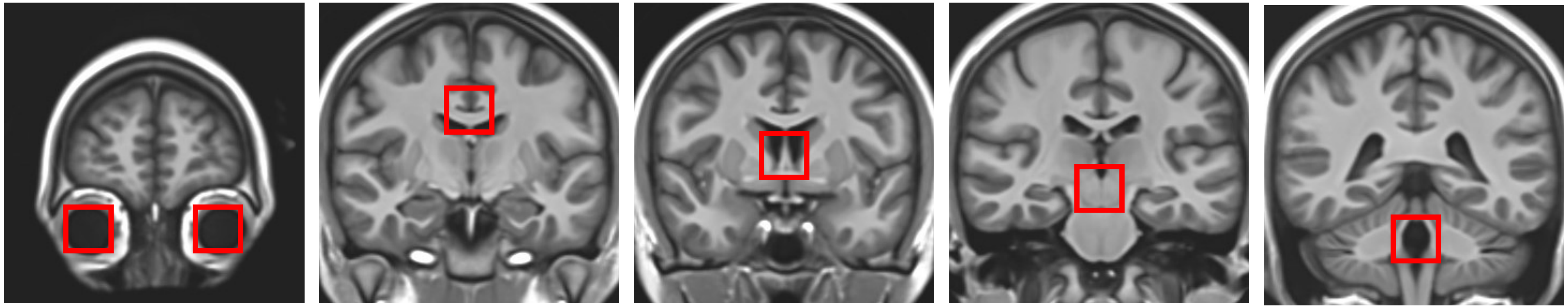

a) Eyes b) Corpus callosum c) Body of fornix d) Midbrain e) 4th ventricle

Supplementary Figure 2 Brain landmarks used for shape normalization, shown in coronal sections. From left to right: eyes, top of corpus callosum, front of body of fornix, center of midbrain, and center of 4th ventricle.

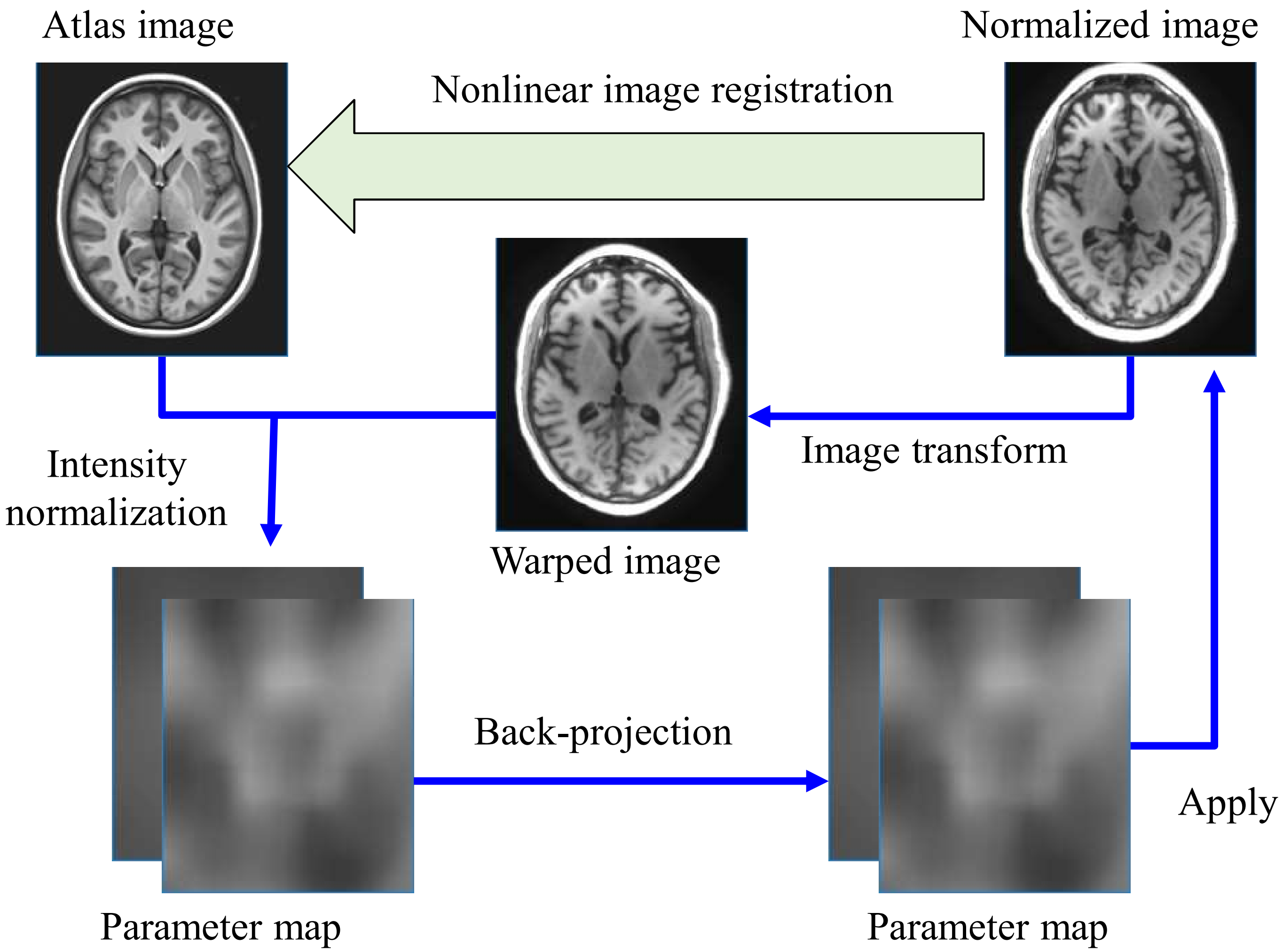

Supplementary Figure 3 Outline of the image intensity normalization algorithm. 1) The shape-normalized image was first aligned to the atlas image. 2) Voxel-wise intensity normalization parameters were calculated for the aligned image. 3) Intensity normalization parameters were back-projected to the shape-normalized image. 4) Intensity of the shape-normalized image was normalized using the back-projected parameters.

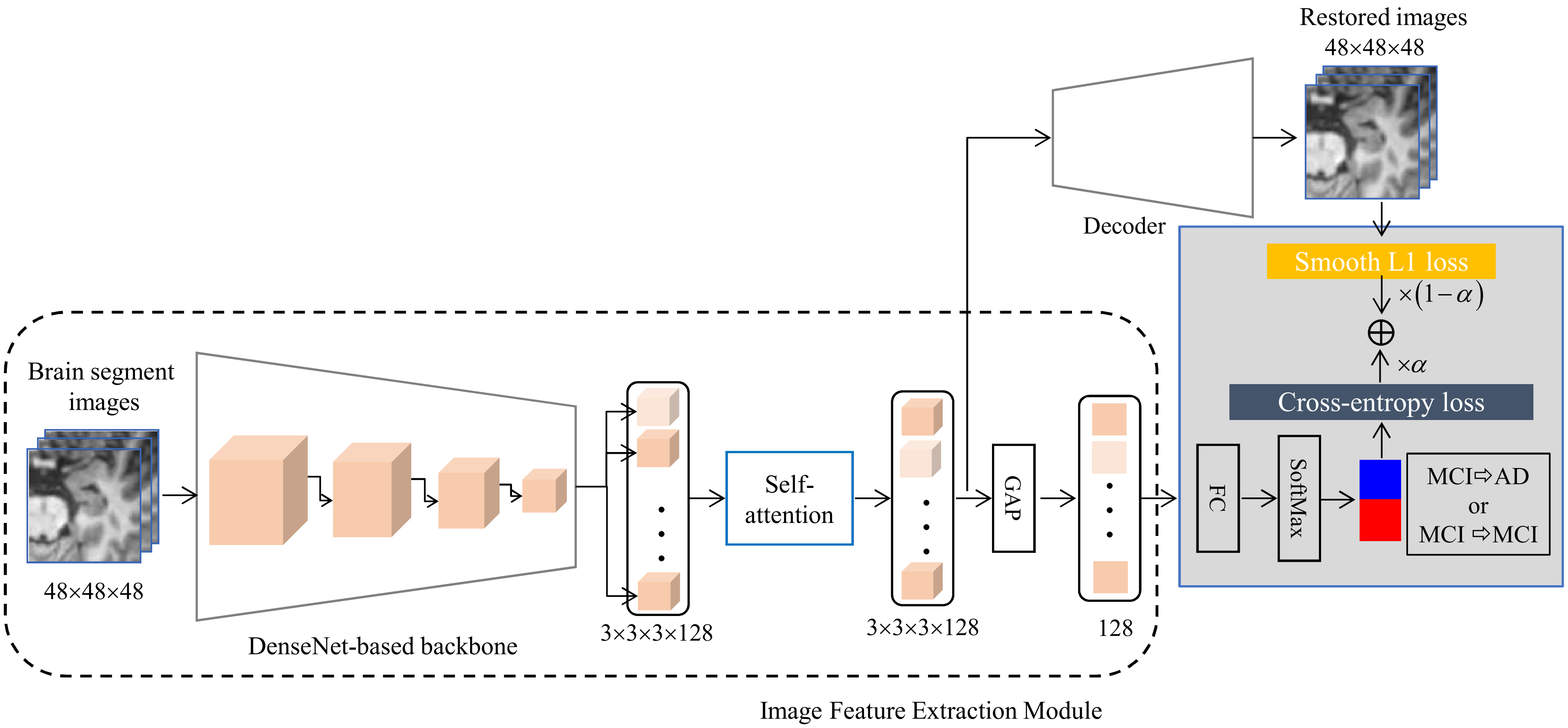

Supplementary Figure 4 The architecture for image feature extraction. The network was based on DenseNet and trained to classify a 48 × 48 × 48 volume to sMCI or pMCI. A self-attention layer was inserted after the last dense block of DenseNet, and a decoder was added to recover the imputed volume using the output of self-attention. Losses of the decoder and classifier were combined with a fixed parameter α, which was set to 0.8. The output of the GAP layer was extracted as image features.

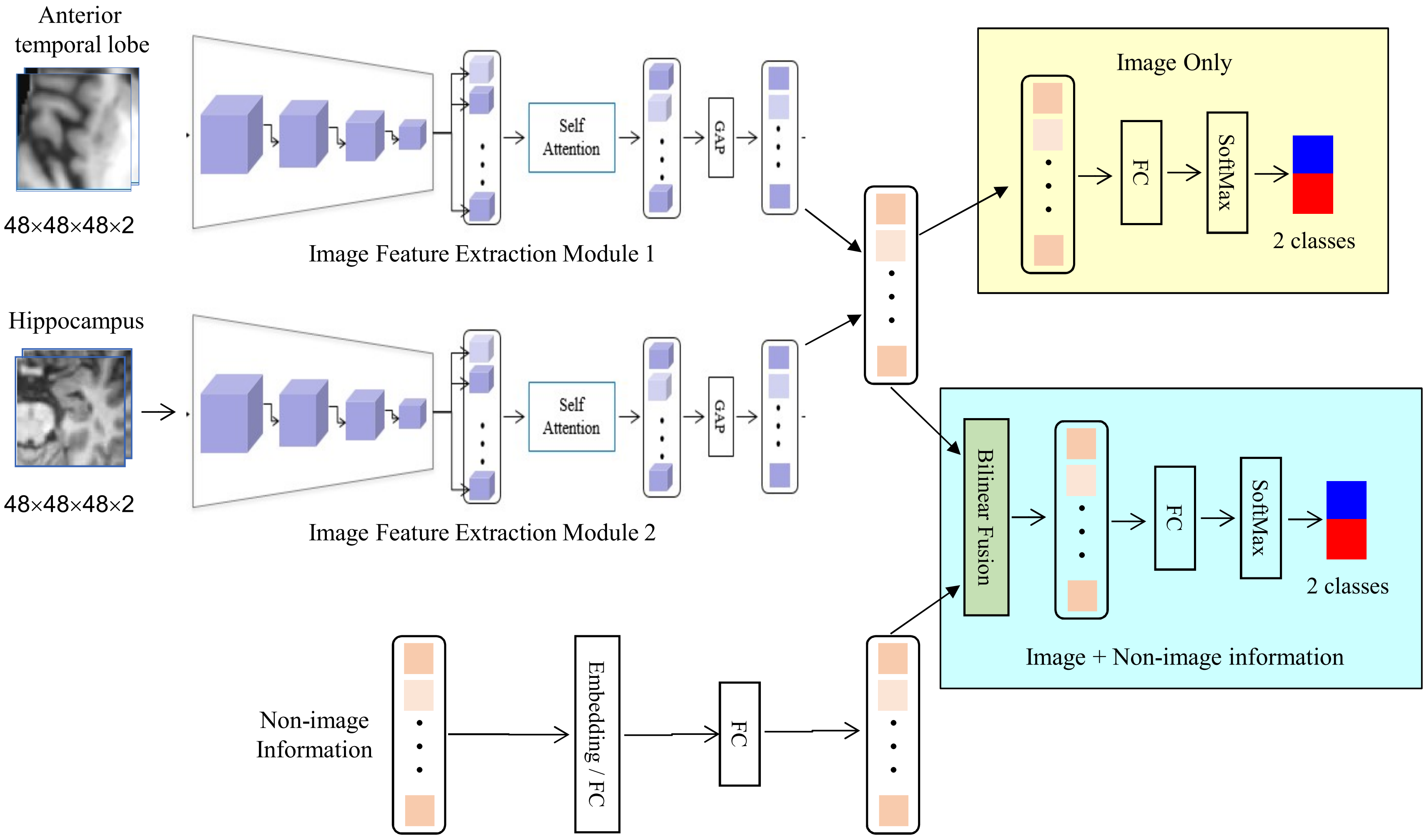

Supplementary Figure 5 The architecture of end-to-end models. For the model using images only, 1) two parallel-configured image extraction modules extract image features from hippocampus and anterior temporal lobe segments; 2) the image features of those segments were then concatenated and input to a classification layer. For the model also using non-image information, non-image information was first extended to the same dimension of the concatenated image features, and bilinear combinations of the concatenated image and extended non-image features were then input to the classification layer.

Supplementary Table 1   Summary of related works. While all the models using only images adopted end-to-end deep learning architectures, most multimodal models used traditional classifiers such as SVM and random forest (RF), except a DNN, which applied a deep survival neural network to a few image features calculated in advance and non-image features, and a DNN that used an end-to-end multimodal deep learning architecture.

Model	Method	Image features used	Clinical features used	Evaluation method
DNN [7]	Multi-scale deep neural networks	volume and mean density of ROI extracted by FreeSurfer	None	10-fold cross validation
DNN [8]	Convolution neural network(CNN)	Whole brain image	None	10-fold cross validation and test
DNN[9]	Densely connected CNN	Whole brain image	None	Validation and test one time
DNN[10]	Residal neural network(RNN)	Grey matter regions extracted by SPM12	None	5-fold cross validation and test repeated 10 times
SVM [11]	Linear SVM and HPS models	Own features using visually clustered 7 subtypes of images	Age, Gender, APoE, MMSE, ADAS	10-fold cross validation
RF[12]	Random forest	Volumes of subregions calculated with MALPEM	Age, Gender	6-fold cross validation, 20 runs
SVM[13]	SVM with RBF kernel	Volume of ROI calculated using BVM and DARTEL	MMSE	Half of samples for validation, fine tuning on validation set
DNN[14]	Deep survival neural network	Volumes of ROI calculated using SPM12 and CAT12	Age, Gender, MMSE	10-fold validation and test, repeated 100 times
DNN[15]	Recurrent neural network (RNN)	Volume of hippocampus, cortical thickness of entorhinal	Age, Gender, APoE, ADNI-EF, ADNI-MEM	5-fold cross validation, repeated 10 times
DNN [16]	CNN with bilinear fusion	Whole brain image	Age, Gender, APoE, MMSE, CDR, FAQ, ADAS	10-fold cross-validation
RF[17]	Random forest	Volume of segments calculated with FreeSurfer	Age, Gender, APoE, , CDR MMSE, ADAS, ADNI_MEM	10-fold cross-validation

Supplementary Table 2 Experimental results of different models. Even only using non-image information, a linear SVM classifier achieved 85% accuracy. Using whole brain images achieved 75% accuracy and was improved to 78% by using brain segments. An end-to-end model using both image segments and non-image information achieved 83% accuracy, which was even lower than that of using only non-image information. A linear SVM using image features extracted from the whole brain by a CNN together with non-image features achieved 87% accuracy and was improved to 88% by using brain segments instead of the whole brain.

Method	Input	Accuracy	AUC	Sensitivity	Specificity
**M0**: Non-image(SVM)	non-image information	0.85	0.93	0.82	**0.88**
**M1**: Whole brain (E2E)	Only image	0.75	0.83	0.70	0.79
**M2**: Brain segments (E2E)	Only image	0.78	0.83	0.78	0.78
**M3**: Brain segments (E2E)	Image + non-image information	0.83	0.92	0.85	0.81
**M4**: Whole brain (SVM)	Image + non-image information	0.87	0.94	0.86	**0.88**
**M5**: Brain segments (SVM)	Image + non-image information	**0.88**	**0.95**	**0.88**	**0.88**

Supplementary Table 3 Cross-cohort evaluation results. An accuracy of 84% and AUC of 91% were maintained for the cohort that was completely unknown to our model.

Method	Input	Accuracy	AUC	Sensitivity	Specificity
Brain segments (SVM)<br>*Trained and tested on NA-ADNI	Image + non-image information	0.88	0.95	0.88	0.88
Brain segments (SVM)<br>*Trained on NA-ADNI and tested on J-ADNI	Image + non-image information	0.84	0.91	0.93	0.71

Supplementary Table 4 Ablation analysis results for model M5. AE contributed the most in the NA-ADNI dataset and SA contributed the most in the J-ADNI dataset. The final model of DenseNet with both AE and SA showed stable performance for both the NA-ADNI and J-ADNI datasets.

Method	Input	NA-ADNI		J-ADNI	
		Accuracy	AUC	Accuracy	AUC
**M0**: SVM	Non-image formation	0.851	0.934	0.808	0.880
**M5.1**: DenseNet+SVM	Image + non-image information	0.873	0.947	**0.838**	0.910
**M5.2**: DenseNet+AE+SVM	Image + non-image information	**0.878**	**0.951**	0.828	0.908
**M5.3**: DenseNet+SA+SVM	Image + non-image information	0.865	0.942	0.833	**0.912**
**M5.4**: DenseNet+AE+SA+SVM	Image + non-image information	**0.878**	0.949	**0.838**	0.909

Supplementary Table 5 Ablation analysis results of model M5 using the RBF kernel for SVM. Compared with Supplementary Table 4, the two models are nearly equivalent, except that the AUCs of the linear model are slightly better.

Method	Input	NA-ADNI		J-ADNI	
		Accuracy	AUC	Accuracy	AUC
**M0**: SVM	Non-image formation	0.847	0.921	0.803	0.858
**M5.1**: DenseNet+SVM	Image + non-image information	0.871	0.938	0.843	0.902
**M5.2**: DenseNet+AE+SVM	Image + non-image information	0.878	0.941	0.838	**0.905**
**M5.3**: DenseNet+SA+SVM	Image + non-image information	0.864	0.934	**0.864**	0.901
**M5.4**: DenseNet+AE+SA+SVM	Image + non-image information	**0.880**	0.941	0.833	0.898

Supplementary Table 6 Variation between validation and test accuracies. M6 is a linear SVM classifier using image features extracted from brain segments. The accuracy gap between the validation and test of hybrid model (**M5**) was much smaller than that of the end-to-end model (**M3**). The same tendency was also seen in the models using images only (**M2** and **M6**). The accuracy varied in a wide range among randomly split validation and test subsets.

Method	Input	Validation/ Test	Mean	Standard Deviation	Minimum	Maximum
**M3**: (E2E)	Image + non-image information	Validation	0.876	0.026	0.847	0.917
		Test	0.832	0.042	0.778	0.903
**M5**: (SVM)	Image + non-image information	Validation	0.886	0.037	0.833	0.931
		Test	0.878	0.034	0.833	0.944
**M2**: (E2E)	Only image	Validation	0.837	0.035	0.778	0.889
		Test	0.778	0.048	0.722	0.861
**M6**: (SVM)	Only image	Validation	0.789	0.037	0.708	0.833
		Test	0.776	0.045	0.694	0.861

Supplementary Table 7 Descriptive statistics from the NA-ADNI and J-ADNI datasets used in our study. MMSE: Mini–Mental State Examination; FAQ: Functional Activities Questionnaire; CDR-SB: Clinical Dementia Rating Sum-Box; ADAS-cog: Alzheimer's Disease Assessment Scale–Cognitive Subscale test.

Category	Stable MCI		Progressive MCI	
	NA-ADNI	J-ADNI	NA-ADNI	J-ADNI
Number (percentage)	399 (55.5%)	80 (40.4%)	430 (44.5%)	118 (59.6%)
Female/Male	159/240	44/36	132/188	69/49
Age (mean ± SD)	73.02 ± 7.63	73.48 ± 5.85	75.86 ± 7.30	74.50 ± 5.57
MMSE (mean ± SD)	27.95 ± 1.77	26.59 ± 1.88	25.92 ± 2.32	24.41 ± 2.14
FAQ (mean ± SD)	1.90 ± 3.09	2.64 ± 2.77	7.47 ± 5.15	7.68 ± 5.31
CDR-SB (mean ± SD)	1.23 ± 0.75	1.44 ± 0.89	2.63 ± 1.10	2.51 ± 1.11
ADAS-cog (mean ± SD)	8.79 ± 3.75	9.44 ± 3.58	14.39 ± 5.45	13.76 ± 4.38

Supplementary Table 8 The specifications of the backbone model of Supplementary Fig. 4. The "conv" operation in the dense block and transition layer contains a sequence of layers of batch normalization, ReLU, convolution, and dropout. The drop rate was set to 0.25 in our experiment. Size in DenseNet121 stands for the filter size for convolution and stride size for pooling.

Layers	Output size	DenseNet121
Convolution	$48 \times 48 \times 48$	$3 \times 3 \times 3\ conv$
Max-pooling	$24 \times 24 \times 24$	$2 \times 2 \times 2\ max - pooling$
Dense Block (1)	$24 \times 24 \times 24$	$\begin{bmatrix} 1 \times 1 \times 1\ conv \\ 3 \times 3 \times 3\ conv \end{bmatrix} \times 6$
Transition Layers (1)	$24 \times 24 \times 24$	$1 \times 1 \times 1\ conv$
	$12 \times 12 \times 12$	$2 \times 2 \times 2\ ave - pooling$
Dense Block (2)	$12 \times 12 \times 12$	$\begin{bmatrix} 1 \times 1 \times 1\ conv \\ 3 \times 3 \times 3\ conv \end{bmatrix} \times 12$
Transition Layers (2)	$12 \times 12 \times 12$	$1 \times 1 \times 1\ conv$
	$6 \times 6 \times 6$	$2 \times 2 \times 2\ ave - pooling$
Dense Block (3)	$6 \times 6 \times 6$	$\begin{bmatrix} 1 \times 1 \times 1\ conv \\ 3 \times 3 \times 3\ conv \end{bmatrix} \times 24$
Transition Layers (3)	$6 \times 6 \times 6$	$1 \times 1 \times 1\ conv$
	$3 \times 3 \times 3$	$2 \times 2 \times 2\ ave - pooling$
Dense Block (4)	$3 \times 3 \times 3$	$\begin{bmatrix} 1 \times 1 \times 1\ conv \\ 3 \times 3 \times 3\ conv \end{bmatrix} \times 16$

Supplementary Table 9 The specifications of the decoder model framework of Supplementary Fig. 4. NBN denotes no batch normalization. “S” denotes up-sampling size.

Input	Operator	Filters	Activation	S
$3^3 \times 128$	deconv3d, $2 \times 2 \times 2$	64	ReLU	2
$6^3 \times 64$	deconv3d, $2 \times 2 \times 2$	32	ReLU	2
$12^3 \times 32$	deconv3d, $2 \times 2 \times 2$	16	ReLU	2
$24^3 \times 16$	deconv3d, $2 \times 2 \times 2$	8	ReLU	2
$48^3 \times 8$	deconv3d, $1 \times 1 \times 1\ NBN$	1	---	1
