# Supplementary material for: A high-generalizability machine learning framework for predicting the progression of Alzheimer’s disease using limited data
Source: NPJ Digit Med. 2022 Apr 12;5:43. doi: 10.1038/s41746-022-00577-x (PMC9005545; doi:10.1038/s41746-022-00577-x)
Supplement: Supplementary file 2 — Reporting summary [file 41746_2022_577_MOESM2_ESM.pdf]

nature portfolio

Corresponding author(s): Caihua Wang, Yuanzhong Li

Last updated by author(s): Dec 2, 2021

Reporting Summary

Nature Portfolio wishes to improve the reproducibility of the work that we publish. This form provides structure for consistency and transparency in reporting. For further information on Nature Portfolio policies, see our Editorial Policies and the Editorial Policy Checklist.

Statistics

For all statistical analyses, confirm that the following items are present in the figure legend, table legend, main text, or Methods section.

n/a	Confirmed	
☐	☒	The exact sample size (n) for each experimental group/condition, given as a discrete number and unit of measurement
☒	☐	A statement on whether measurements were taken from distinct samples or whether the same sample was measured repeatedly
☐	☒	The statistical test(s) used AND whether they are one- or two-sided<br>*Only common tests should be described solely by name; describe more complex techniques in the Methods section.*
☒	☐	A description of all covariates tested
☐	☒	A description of any assumptions or corrections, such as tests of normality and adjustment for multiple comparisons
☐	☒	A full description of the statistical parameters including central tendency (e.g. means) or other basic estimates (e.g. regression coefficient) AND variation (e.g. standard deviation) or associated estimates of uncertainty (e.g. confidence intervals)
☒	☐	For null hypothesis testing, the test statistic (e.g. F, t, r) with confidence intervals, effect sizes, degrees of freedom and P value noted<br>*Give P values as exact values whenever suitable.*
☒	☐	For Bayesian analysis, information on the choice of priors and Markov chain Monte Carlo settings
☒	☐	For hierarchical and complex designs, identification of the appropriate level for tests and full reporting of outcomes
☒	☐	Estimates of effect sizes (e.g. Cohen's d, Pearson's r), indicating how they were calculated

Our web collection on statistics for biologists contains articles on many of the points above.

Software and code

Policy information about availability of computer code

Data collection	The code of DenseNet which was used as the backbone of our architecture is available at https://github.com/liuzhuang13/DenseNet. The code of Faster R-CNN used for brain landmark detection is available at https://github.com/rbgirshick/py-faster-rcnn. Insight Toolkit: ITK on which our image registration algorithm was implemented is available at https://itk.org
Data analysis	Data analysis used Python v3.7(https://www.python.org/), NumPy v1.17 (https://github.com/numpy/numpy), SciPy v1.2.1 (https://www.scipy.org/), seaborn v0.11.1 (https://github.com/mwaskom/seaborn), pandas v1.1.5 (https://github.com/pandas-dev/pandas), tensorflow v1.15.0(https://www.tensorflow.org/), scikit-learn v1.0(https://scikit-learn.org/)

For manuscripts utilizing custom algorithms or software that are central to the research but not yet described in published literature, software must be made available to editors and reviewers. We strongly encourage code deposition in a community repository (e.g. GitHub). See the Nature Portfolio guidelines for submitting code & software for further information.

Data

Policy information about availability of data

All manuscripts must include a data availability statement. This statement should provide the following information, where applicable:

- Accession codes, unique identifiers, or web links for publicly available datasets
- A description of any restrictions on data availability
- For clinical datasets or third party data, please ensure that the statement adheres to our policy

All input data are freely available from public sources.
The data used for model training, validation and testing are publicly available at following URL. 1. NA-ADNI dataset: http://adni.loni.usc.edu/ 2. J-ADNI dataset: https://humandbs.biosciencedbc.jp/en/hum0043-v1

Field-specific reporting

Please select the one below that is the best fit for your research. If you are not sure, read the appropriate sections before making your selection.

☒ Life sciences ☐ Behavioural & social sciences ☐ Ecological, evolutionary & environmental sciences

For a reference copy of the document with all sections, see nature.com/documents/nr-reporting-summary-flat.pdf

Life sciences study design

All studies must disclose on these points even when the disclosure is negative.

Sample size	No sample size was chosen; all the samples in ADNI and J-ADNI datasets meets our definition, that is, patients diagnosed as mild cognitive impairment (MCI) at a baseline time, with T1 wighted MRI image and cognitive test scores of MMSE, CDR, FAQ and ADAS-cog available at the baseline time, either converted to Alzheimer's Disease (AD) in two years or stayed as MCI till two years from the baseline time, were used in our study.
Data exclusions	T1 wighted MRI scans with resolution (voxel size) > 1.2mm was excluded from our study.
Replication	Not applicable, no experimental work is described in this study. Because our method is a hybrid one using DenseNet and SVM which are available in Open Sources listed in Software and code, and the data is available publically, the results can be replicated.
Randomization	Not applicable, we are not making a comparison between two groups
Blinding	Not applicable, we are not making a comparison between two groups

Reporting for specific materials, systems and methods

We require information from authors about some types of materials, experimental systems and methods used in many studies. Here, indicate whether each material, system or method listed is relevant to your study. If you are not sure if a list item applies to your research, read the appropriate section before selecting a response.

Materials & experimental systems

n/a	Involved in the study	
☒	☐	Antibodies
☒	☐	Eukaryotic cell lines
☒	☐	Palaeontology and archaeology
☒	☐	Animals and other organisms
☒	☐	Human research participants
☒	☐	Clinical data
☒	☐	Dual use research of concern

Methods

n/a	Involved in the study	
☒	☐	ChIP-seq
☒	☐	Flow cytometry
☒	☐	MRI-based neuroimaging
